# Intensification of ice nucleation observed in ocean ship emissions

**DOI:** 10.1038/s41598-018-19297-y

**Published:** 2018-01-18

**Authors:** E. S. Thomson, D. Weber, H. G. Bingemer, J. Tuomi, M. Ebert, J. B. C. Pettersson

**Affiliations:** 1 Atmospheric Science, Department of Chemistry and Molecular Biology, University of Gothenburg, Gothenburg, 41296 Sweden; 2 Institute for Atmospheric and Environmental Sciences, J. W. Goethe-University, Frankfurt am Main, Frankfurt, 60438 Germany; 3 Institute for Applied Geosciences, Technical University of Darmstadt, Darmstadt, 64287 Germany

## Abstract

Shipping contributes primary and secondary emission products to the atmospheric aerosol burden that have implications for climate, clouds, and air quality from regional to global scales. In this study we exam the potential impact of ship emissions with regards to ice nucleating particles. Particles that nucleate ice are known to directly affect precipitation and cloud microphysical properties. We have collected and analyzed particles for their ice nucleating capacity from a shipping channel outside a large Scandinavia port. We observe that ship plumes amplify the background levels of ice nucleating particles and discuss the larger scale implications. The measured ice nucleating particles suggest that the observed amplification is most likely important in regions with low levels of background particles. The Arctic, which as the sea ice pack declines is opening to transit and natural resource exploration and exploitation at an ever increasing rate, is highlighted as such a region.

## Introduction

Cloud radiative properties, precipitation, and cloud electrification all respond to and are largely controlled by the formation and evolution of cloud ice. Ice Nucleating Particles (INP) are those atmospheric particles, which lower the free energy barrier that exists to spontaneous nucleation^1^. Mineral dust, biological particles, and other primary particles have all been identified as ice nucleators, yet the abundance of such INP does not match the observed macroscopic features of clouds and precipitation^2–5^. Although, some mechanisms of ice multiplication are known^6–8^, the “closure” between abundance and composition of cloud-active aerosol, cloud ice, and precipitation remains a significant scientific hurdle.

It is thought that globally mineral dust particles are the most common INP^9^, and they have therefore been used as a proxy for calculating global INP climatologies^10^. Laboratory studies of soot particles acting as INP have been more ambiguous, with many investigators failing to find any measurable nucleating capabilities above homogeneous freezing temperatures^11–14^. That said it is known that soot has a morphology that evolves considerably with aging, as can surface absorbents and therefore hygroscopicity. There is some evidence that increased hygroscopicity will lead to more favorable ice nucleation conditions^15^, and field measurements of aged and mixed soot have shown it to be an active ice nucleator^16,17^. Further complicating the picture is that there is not necessarily a strong link between liquid condensation and ice nucleation^18^, and some soot-surface coatings may act to suppress ice formation^19^.

In polar regions, which are some of the most remote and pristine areas of the world, what dominates ice nucleation may also be unique. As a deep ocean covered by a thin veneer of ice, the Arctic in particular is a unique and important area that demonstrates sensitive responses to cloud and radiative feedbacks^20,21^. For most of the year the Arctic is well isolated from the middle and lower latitudes and is thus characterized by very low particle concentrations^22^. From May through September nearly 70% of the central Arctic is covered by low-level stratiform clouds^23^ -the radiative properties of which depend upon their microphysics^24^. Thus small changes in terms of regional sources and/or large scale transport of particulate may have significant impacts^25^. Furthermore, in the Arctic the sea-ice albedo feedback is highly sensitive to the radiative forcing of clouds^21^, and mixed-phase systems, in which solid ice and liquid water sustain coexistence, predominate – suggesting changes in INP may have amplified environmental effects. Therefore, it is important to understand the physical and chemical characteristics of INP that contribute to Arctic climate, and to its potential perturbation.

**Figure 1:**
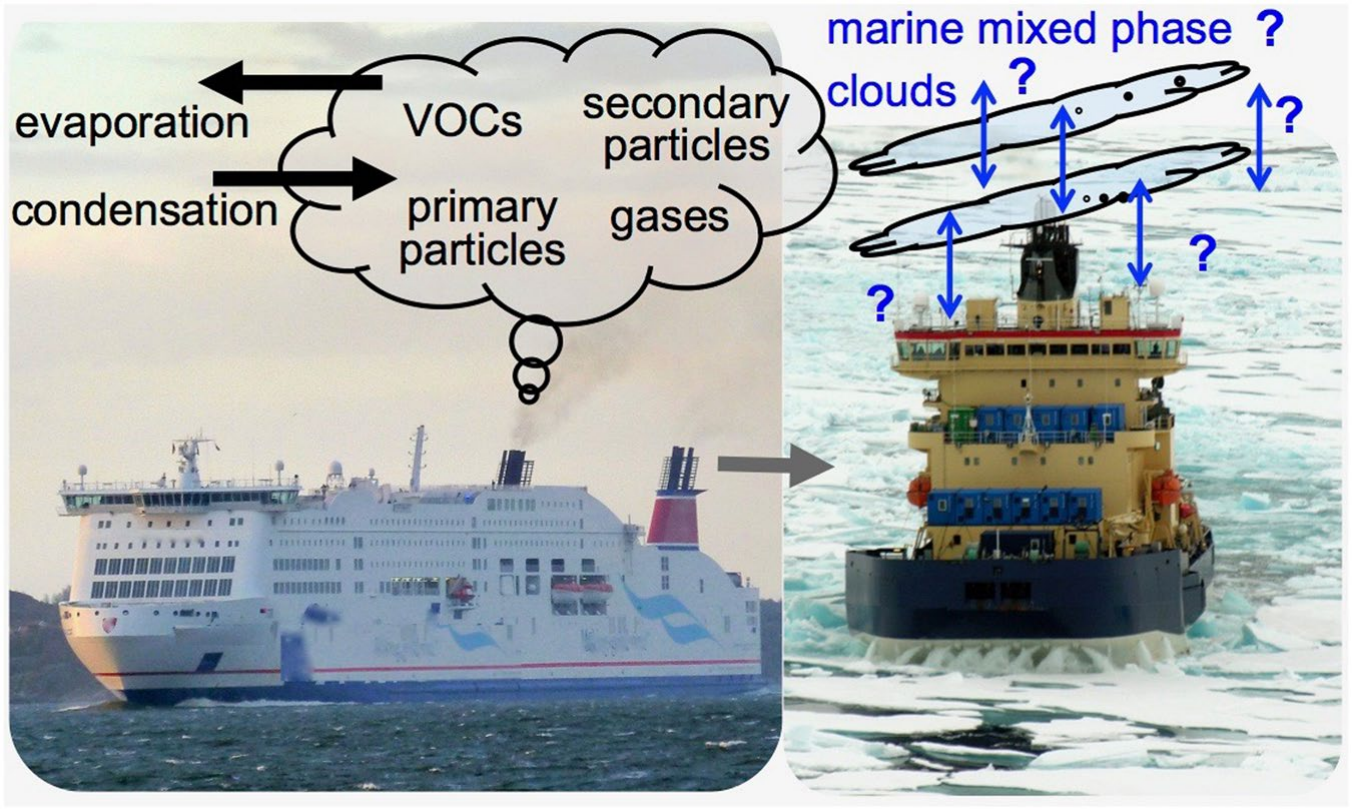
Shipping contributes a range of gas and particle emissions to the aerosol burden that may be more acutely felt in sensitive ecosystems. The left hand image of a passenger ferry taken from measurement site at Risholmen penninsula (Fig. 2) illustrates the ship’s exhaust plume, which consists of primary gases and particles and can transform vis-á-vis a number of chemical and physical processes. The icebreaker image (right, courtesy of K. Abrahamsson), illustrates the link to marine mixed phase clouds, that may be uniquely sensitive to emissions in areas of more pristine aerosol.

Arctic shipping, including maritime natural resource exploration and exploitation, is an area of both economic growth and an obvious source of anthropogenic particles and pollutants (Fig. 1)^26^. At the turn of the millennium polar shipping was dominated by community re-supply, fishing, and tourism^27^, but future projections foresee increases in both destination traffic^28^, and also an inevitable push towards large cargo transit shipping as navigable northern maritime routes open due to changes in climate and therefore sea ice extent^29,30^. The northern Europe-Asia routes can be significantly shorter, and therefore save considerable fuel (the major cost in international shipping) relative to the traditional Suez Canal passages. Shorter transit distances and lower fuel consumption are in general positive for the global shipping footprint, but not all locations are created equal and the Arctic is a particularly unspoiled ecosystem^31^. An even stronger impact will be made by oil and gas shipping, which typically has 50% higher fuel consumption than transit shipping^27^. The arrival of more shipping in the Arctic basin is inevitable if even the most optimistic climate projections are correct, but the climate impact and feedbacks are not so certain. Although, many of the primary gas and particle emissions are already studied and it is known that many of them like black carbon have both long and short-lived climate forcing effects, specific effects of INP on clouds and climate have not been fully considered. For example, using the INP observations we present, Possner *et al*.^32^ recently simulated stratiform mixed-phase cloud response to INP emissions^32^. They show that moisture content, precipitation, and cloud-top radiative feedback all respond in non-linear, a-systematic ways, that can be sensitive to the cloud history and background state.

Herein we present the results of ship plume measurements made in 2013 and 2014 in a Scandinavian shipping channel. The results demonstrate the ability of ship emissions to amplify the number of atmospheric INP. The results are compared to background conditions to illustrate that even low absolute levels of introduced INP may contribute significantly in relatively pristine areas. The implications for Arctic clouds and climate are discussed.

## Measurements

**Figure 2:**
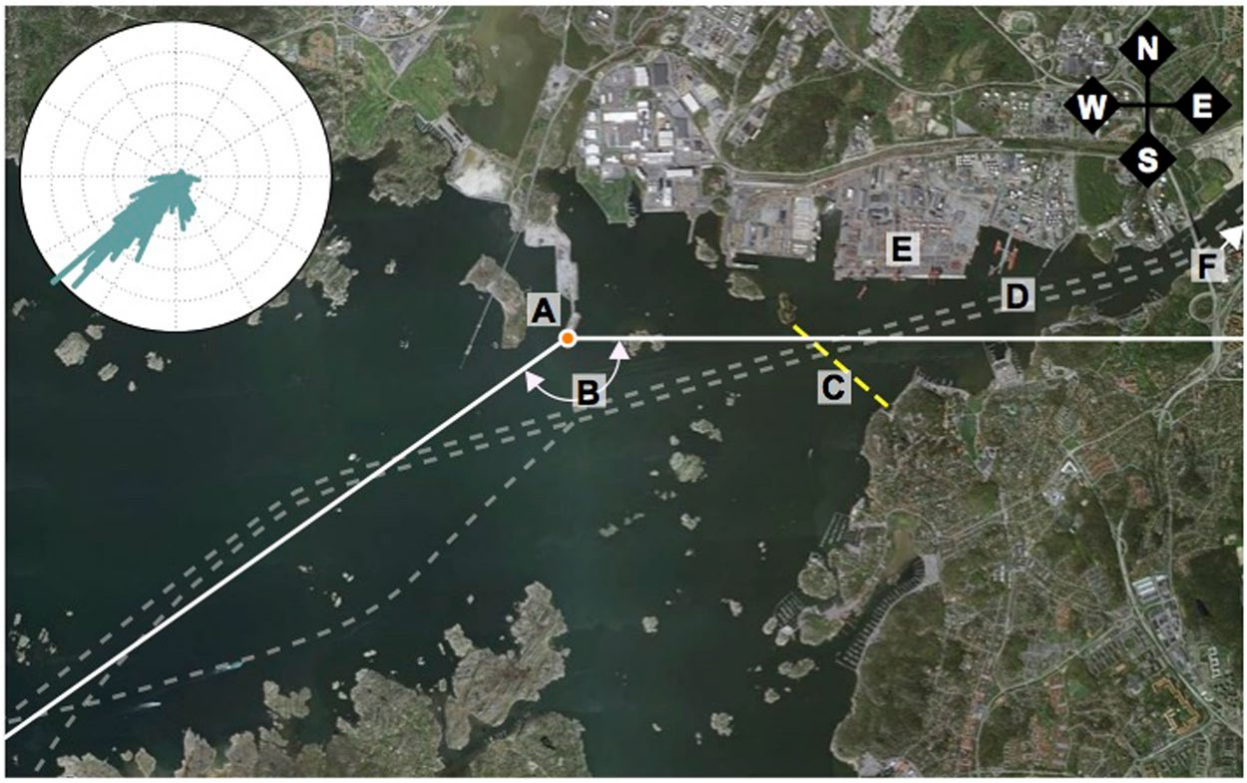
Ariel view of the Port of Gothenburg and Risholmen measurement site. (A) Sampling location at the tip of Risholmen peninsula (B). Viable range for plume sampling from ≈90° to ≈235°, (C). Inner port speed limit boundary (8 knots) (D). Shipping lanes (E). Container terminal (F). Ferry terminals and inner port (outside map boundary). At the upper left corner a wind rose displays the wind directions measured during 2013 INP collection days. Map created using Google Earth Pro (7.1.4.1529), Image © 2016 DigitalGlobe.

The Port of Gothenburg is a river and deep water sea port and is the largest Scandinavian cargo port with approximately 11,000 vessel calls annually. The port’s container, cargo, and ferry terminals all lie well inside the Gothenburg archipelago, where a relatively narrow shipping lane threads between many islands and peninsulas. The measurements described here were made from the Risholmen peninsula (57.68370°N, 11.80450°E, Fig. 2) on the north side of the primary cargo and passenger shipping channel and seaward of both the mouth of the Göta Älv river and the inner harbor speed limited zone. The Port of Gothenburg straddles the International Maritime Organization (IMO) Baltic and North Sea Emissions Control Areas (ECAs), where during the 2013 and 2014 measurement campaigns fuel sulfur content (FSC) was limited to 1% by mass^33,34^. Outside of the ECAs the IMO regulatory limit on FSC is currently 3.5% and will drop to 0.5% in 2020. Thus, the Risholmen location allows for measurements of plumes from ships operating under near open water conditions and that largely comply with the future regulatory structures of global shipping.

**Figure 3:**
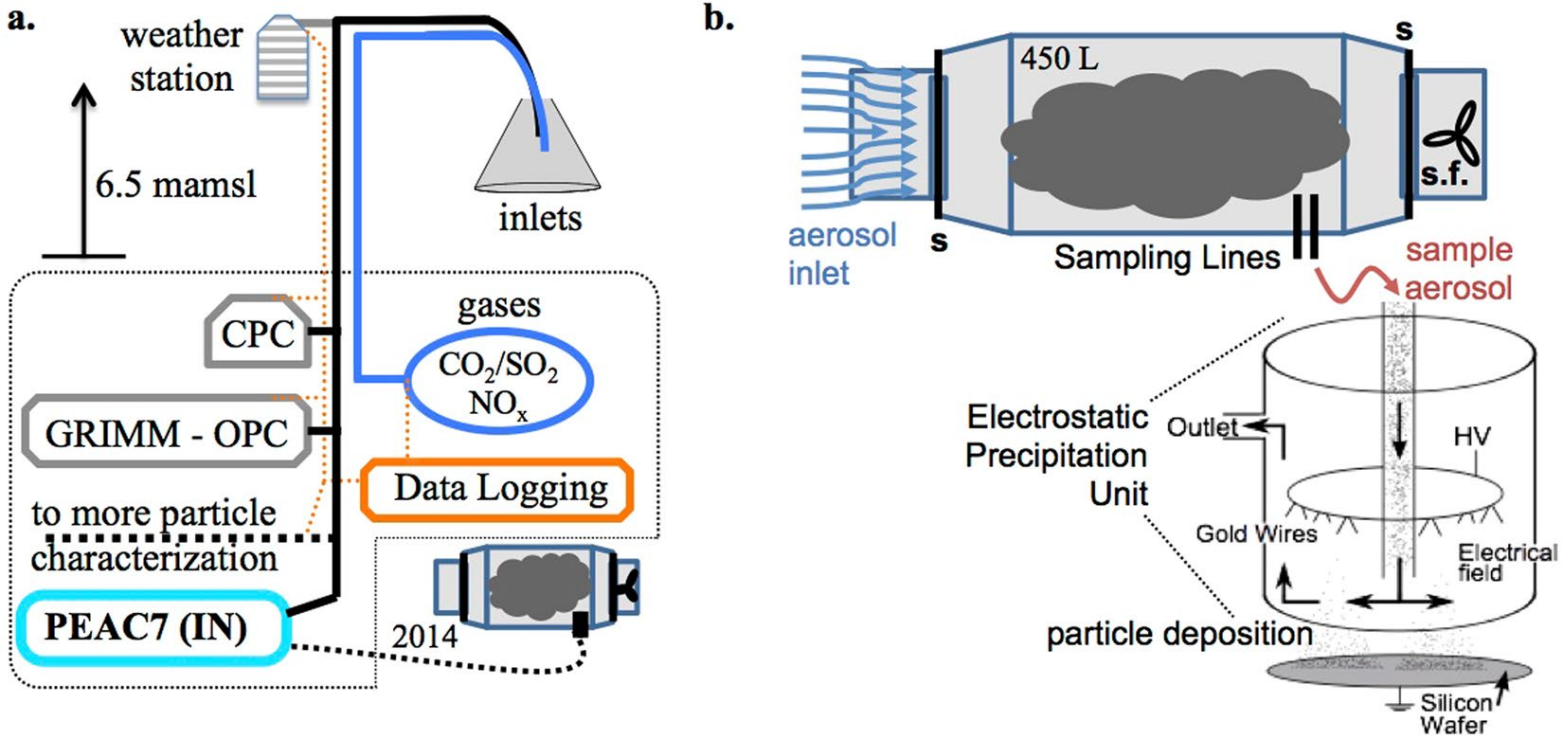
Schematic of the plume sampling instrumentation pertaining to INP collection. (**a**) Particle and gas inlets mounted 6.5 meters above sea level led to various instrumentation more completely described in the text including the PEAC7 INP collector. (**b**) For the 2014 field season the particle collection protocol was altered to include a plume capture chamber. The **s** symbols represent the stopping valves and **s.f**. indicates the location of the sucker fan. The internal functions of the PEAC7 electrostatic deposition unit are detailed.

From 2013–2015 annual measurement campaigns were undertaken at Risholmen that focused on the physical and chemical characteristics of ship plume gas and particle emissions^35^. Ship plumes were sampled intermittently as ships arrived to and departed from the Port of Gothenburg and thus passed the measurement station. Measurements included meteorology, gas monitoring, particle counting (0.1 *μ*m to 10 *μ*m), soot characterization, gas and particle mass spectrometry, cloud condensation nuclei (CCN), and in 2013 and 2014 INP characterization (see Fig. 3). For the data presented here the INP were collected using a programmable PEAC7 electrostatic deposition unit that has been previously summarized^36^. The PEAC7 charges aerosol particles in a flow of sample air and subsequently precipitates the particles onto the surface of a grounded silicon wafer disc. These sample substrates were returned to a laboratory and were analyzed using the Frankfurt isothermal static diffusion chamber for ice nucleation FRIDGE^36,37^. FRIDGE is an instrument designed for activation, growth and counting of the INP collected on the silicon wafer substrates. Additional analysis of the semi-conducting silicon wafers can also be done using scanning electron microscopy (SEM) and other methods in order to chemically and physically characterize captured particles.

The prevailing meteorology during INP sampling varied between ship passages but was generally representative of late-fall Nordic maritime climatic conditions. Diurnal variability was limited by the strong marine influence, resulting in conditions dominated by the passage of frontal cyclones. During INP sampling wind speeds typically ranged from 3–13 m/s with temperatures from 3–12 °C. Relative humidities at the sea shore stayed high (60–85%) with periods of fog and rain. Plume travel times between 30 seconds and 3.5 minutes can be estimated from the wind direction/speed and the distance from the shipping channel. Given the dominant wind vectors most plumes sampled for INP had travel times at the lower end of this interval (30–60 s.), where ships were sampled when approaching to within 300 meters of the station.

### Particle Collection

Aerosol particles were sampled for INP at the Risholmen site over the course of two field campaigns during the fall of 2013 and the fall of 2014. In 2013 ship plume particles were collected from individual ships onto wafers using the PEAC7 sampling unit^36^. The sample collection proceeded directly via a particle inlet for the time period that the individual plumes transited the sampling site. The presence of plume particles was anticipated using ship tracking software, with the plume arrival detected by measuring particle counts using a CPC (Condensation Particle Counter, Model 3775, TSI Inc., Fig. 3(a)). Sampling with the PEAC7 at 2 L/min was initiated immediately upon seeing a sharp upward inflection in the realtime CPC count intensity. The transit time of plumes at the measurement site depended upon wind speed and direction and ranged from 2–10 minutes, with sampling continuing 30–60 s after the CPC count magnitude returned to stable background levels.

Subsequent to the 2013 measurements the deposition wafers were analyzed and yielded measurable but low absolute INP counts. In an effort to increase statistical reproducibility the results of 2013 prompted an updated 2014 sampling protocol to increase the total sampling volume and thus the absolute number of collected INP irrespective of the plume transit time. Thus in 2014 a 450 L plume capturing chamber (Fig. 3) was used to capture the transient plumes and allow for sampling times of up to 25 minutes. The plume capture chamber consisted of large tubular sections of galvanized steel tubing. At each end of the tubing 10 cm diameter stopper valves were mounted, with a sucker fan mounted behind the exit valve. The sampling proceeded by continuously running the fan until the plume arrival was detected using the CPC count intensity. When the CPC count peaked the fan was turned off and the stopper valves were closed, isolating a volume of the plume within the 450 L chamber. The PEAC7 was used to sample the captured plume through sampling ports in the closed chamber with a flow rate set to ≈4 L min^−1^, and independently measured for each collection interval. An EEPS (Engine Exhaust Particle Sizer, Model 3090, TSI Inc., 5.6–560 nm) and an optical particle counter (≥0.3 *μ*m, dust monitor model 1.108, GRIMM Labortechnik GmbH & Co.KG) were used to characterize particles within the capturing chamber. Measurements showed stable particle size distributions for ≈30 minutes at 10 L/min sampling flows, confirming that INP sampling losses from sedimentation and/or diffusion to the chamber walls were negligible during PEAC7 sampling increments. Fifty to sixty litres of air were sampled for each wafer in 2014.

In both measurement years, following plume sampling, background air samples were collected in an identical manner to the plume samples. Thus each ship plume sample is paired with a background sample taken under equivalent meteorological conditions and using the same sampling parameters. The separate but contiguous INP sampling used here makes it unique for INP studies, and with differencing allows an enhancement factor to be calculated from the data.

### Wafer Analysis

The FRIDGE static diffusion system consists of a chamber where sampling wafers are exposed to controlled temperature and H_2_O vapor pressure conditions. The wafers are monitored using an optical CCD system that is interfaced with a computer and image analysis software, and allows individual grains of nucleated ice to be identified and counted. The system has previously been described in detail^37^ and recently Schrod *et al*.^36^ have amended and confirmed the robustness of the measurement technique.

The sample wafers collected at the Risholmen measurement site were individually analyzed at nine temperature T and ice supersaturation S_*i*_ conditions as reported in Table 1. Additionally, a clean wafer was analyzed at each of the T and S_*i*_ conditions to measure a ‘blank’ value. The number of INP per unit volume of air is calculated based upon the counted INP and the recorded sampling parameters as recommended by Schrod *et al*.^36^.

**Table 1:** Temperature and saturation conditions used for FRIDGE analysis.

Temperature (K)	255	255	251	251	248	248	248	243	243
S*i*	1.15	1.18	1.20	1.22	1.19	1.22	1.26	1.29	1.32

## Results and Analysis

**Figure 4:**
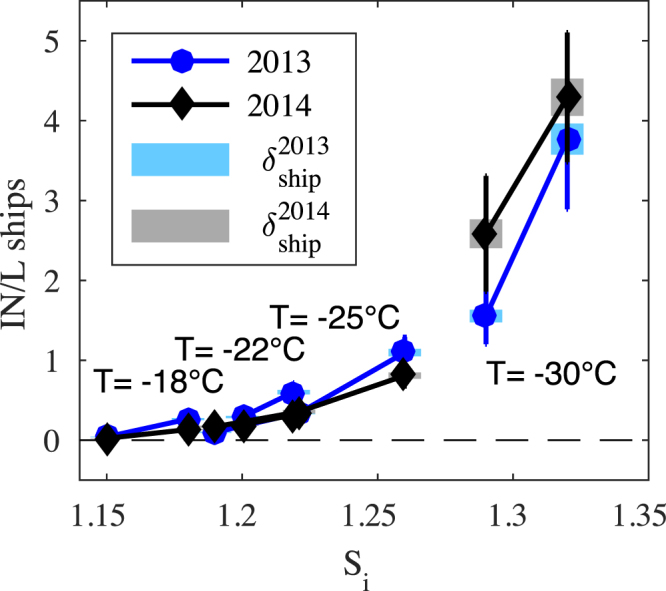
Mean INP concentration measured for all ships in campaign years 2013 and 2014 as a function of supersaturation with respect to ice. The supersaturations are achieved at the temperatures indicated and precisely specified in Table 1. Solid error bars represent the standard error of the mean and the shaded bars represent the uncertainty representative of single ship measurements.

The mean absolute number concentrations (INP L^−1^) measured by the FRIDGE apparatus for all ships in 2013 and 2014 are shown in Fig. 4. In 2013, 53 ship plumes were sampled for INP from a range of ship classes including passenger ferries, cargo ships, and gas/oil tankers. In 2014 another 30 ships were sampled across classes. The data are presented with two error envelopes to represent, (i) the uncertainty in any single INP measurement (shaded bars), and (ii) the deviation between the ship measurements in a single measurement season (solid, vertical bars)^36^. Thus the standard error of the mean given by the solid bars is indicative of the large ship-to-ship variation in emitted INP. In both years the variability between individual ships far outweighed any identifiable trends based on ship type, and as can be seen from the overlapping solid error bars in Fig. 4 the ship-to-ship variability also encompasses the variability between the two sampling campaigns. Although the background INP concentrations also exhibit variability for the Gothenburg harbor conditions they are typically ≤50% of those measured within the plumes. Figure 4 also confirms that the 2014 measurements made using the plume capture chamber are entirely consistent with the directly sampled plume data from 2013.

Although it might be expected that secondary (aging) processes could influence the ice nucleation ability of ship emissions no significant correlations between INP concentration and ambient conditions were observed. This suggests that the short plume travel time and variable but narrowly constricted ambient conditions did not impact the results. The effect of plume aging remains an open scientific question that should be further investigated.

**Figure 5:**
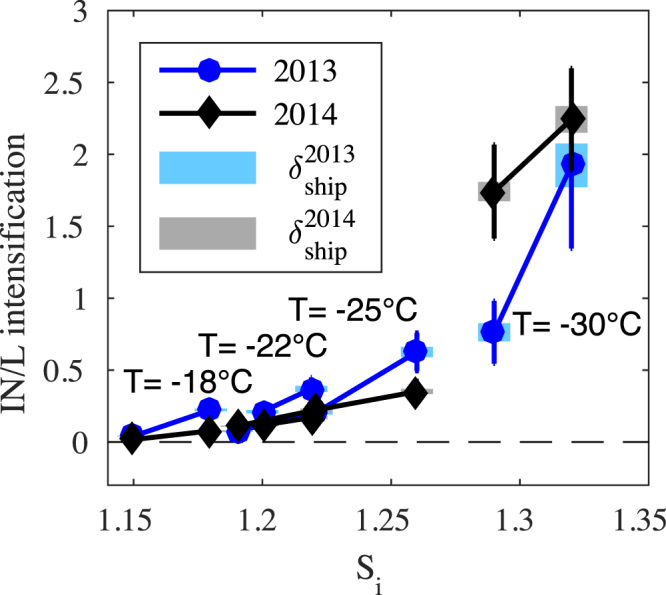
INP enhancement observed within shipping plumes. As in Fig. 4 the supersaturations are achieved at the temperatures indicated and precisely specified in Table 1. Solid error bars represent the standard error of the mean and the shaded bars represent the uncertainty representative of single ship measurements.

The observed effect of ship emissions on INP is shown in Fig. 5 where the average increase of INP concentration (INP L^−1^) above the measured background state is plotted for both 2013 and 2014. The intensification is taken to be the difference in concentration ${\rm{\Delta }}\mathrm{INP}={\overline{INP}}_{{\rm{ship}}}-{\overline{INP}}_{{\rm{bk}}}$, between the mean INP concentration measured in the plumes ${\overline{INP}}_{{\rm{ship}}}$, and the mean INP concentration from the equivalent background measurements ${\overline{INP}}_{{\rm{bk}}}$, where the uncertainties are also propagated. In both years an enhanced abundance of INP is observed due to particles captured from the ship plumes. The effect is small at high temperatures and low supersaturations but strengthens considerably as the thermodynamic driving force grows. Furthermore, a comparison of Figs 4 and 5 shows that although a range exists, the INP enhancement is of order and can even exceed the background signal.

In order to extrapolate from these land-based, stationary measurements, emission factors *EF* may be used. An INP emission factor *EF*_INP_ relates the number of emitted INP to the total mass of fuel comsumed. For combustion plumes CO_2_ concentration has been shown to be a reliable tracer of fuel consumption and thus can be used as a basis for calculating gas and particle emissions as a function of mass of fuel burned^38^. Such calculations also depend on the conversion efficiency of the hydrocarbon fuel to CO_2_, where here we assume the mass of emitted CO_2_ per kg fuel burned is $E{F}_{{{\rm{CO}}}_{2}}=\mathrm{3.2\ }{\rm{kg}}{({\rm{kg}}{\rm{fuel}})}^{-1}$^39,40^. Thus given $E{F}_{{{\rm{CO}}}_{2}}$, *EF*_INP_ is determined by the ratio between the increase of INP concentration ΔINP to the corresponding increase of CO_2_ referenced to standard temperature and pressure ΔCO_2_,

1 $$E{F}_{{\rm{INP}}}{({\rm{kg}}{\rm{fuel}})}^{-1}=\frac{{\rm{\Delta }}\mathrm{INP}}{{{\rm{\Delta }}\mathrm{CO}}_{2}}\times E{F}_{{{\rm{CO}}}_{2}}\mathrm{.}$$

During both measurement campaigns CO_2_ concentration, temperature, and local pressure were measured continuously with one second time resolution. Thus ΔCO_2_ could be straightforwardly calculated by integrating the plume signal minus the background concentration.

**Figure 6:**
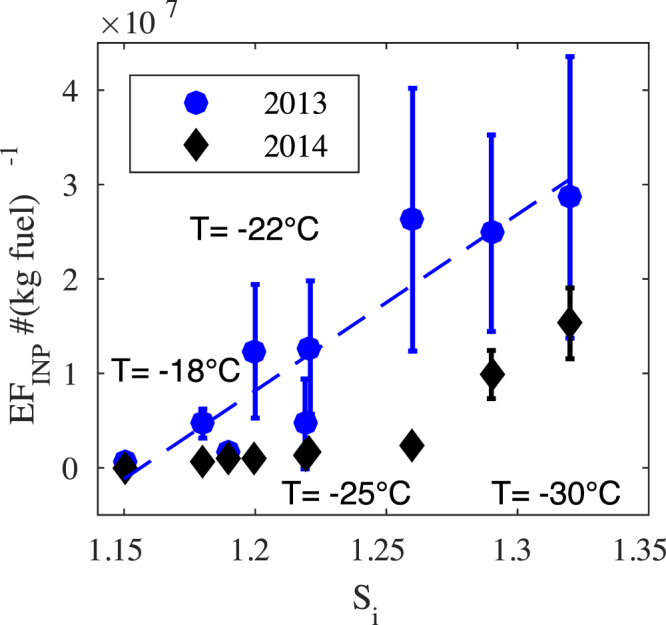
Emission factors for INP emitted from ship plumes with error bars indicating the standard error.

Emission factors were calculated for individual ships at each T and S*i* condition and averaged as in Figs 4–5. The results which range in magnitude from ≥10^5^ to ≥10^7^ are plotted in Fig. 6 accompanied by the standard error of the mean and a linear least squares fit to the 2013 data. It is difficult to determine whether the differences between the data represent significant changes from 2013 to 2014, but given the generally close overlap it is unlikely the differences will have a big effect on the utility of the findings. It is important to note that the calculated INP emission factors are small compared to the total particle emission factors which for this study $\approx {\mathscr{O}}{\mathrm{(10}}^{17}{({\rm{kg}}{\rm{fuel}})}^{-1})$ compare favorably with previous studies^38^.

One advantage of utilizing the FRIDGE technique is that subsequent to INP analysis the deposition wafers can be analyzed using SEM to study chemical composition, size and morphology of single particles. A SEM analysis of a limited set of 2013 wafers was done using energy-dispersive x-ray spectroscopy (EDX), with the aim of qualitatively evaluating the chemical composition of particles acting as INP. Fifty-one INP positions, identified from the FRIDGE image analysis^36^, were investigated on four different wafers. From the background measurements 13 of 15 particles had substantial carbon (16–79 wt%) and oxygen content (6–81 wt%), which may be indicative of oxygen associated with carbonaceous material. However, more than 50% (8 of 15) had an O/C ratio above 0.5 and/or a substantial inorganic content (Fe, Ca, Mg, Al, Mo, Na, K and/or Cl), and thus inorganic phases like mineral dust and even sea salt appear to be present in many background INP. Conversely, of the analyzable single particles, those from the shipping wafers were only identified as soot.

## Discussion and Atmospheric Implications

We see two interesting results from this study. First we do observe ice nucleation from ship plume particles that are most likely to be fresh soot particles. Given that the cursory single particle analysis on 2013 wafers pinpointed only unremarkable carbonaceous soot particles, no exhaustive particle characterization was completed. However, it is not unforeseen that some subset of soot particles do have nucleating capacity, and given the FRIDGE’s ability to detect low concentrations due to the flexibility of the sampling protocol, we see the result manifested here. This is not entirely at odds with previous measurements of the ice nucleating potential of combustion particles, although previously observations of combustion soot nucleation have largely been limited to the homogeneous regime, or where soot particles only act as freezing nuclei after activating liquid droplets^11,15,18^. Some such results may be attributed to experimental resolution, as many studies demonstrate that fresh, laboratory generated soot is weakly ice nucleating. Schill *et al*.^14^. observed activated fractions from off-road diesel emissions of between 10^−6^ and 10^−8^. Likewise, maximum activated fractions of ≤10^−5^ have been observed for automobile diesel emissions^41^, which are similar to those observed from a propane diffusion flame (≤10^−4^)^13^. Dymarska *et al*.^12^ posit an upper limit for ice production from a range of experimentally measured soot types that corresponds to an activated fraction of ≈0.5 × 10^−6^. While these low magnitudes have largely been dismissed as unlikely to be important on a global scale, our measurements demonstrate that within the context of a low background of INPs – such point sources may notably shift the ambient condition.

Second, we identify another potential indirect cloud and climate effect that should be considered for ship emissions in pristine environments. Attempts to quantify INP in polar regions have been complicated by instrument resolution, but the available records indicate low concentrations of ambient INP on the order of one per liter (Table 2). Clearly, the scatter of measured Arctic INP abundance is large, making strict quantitative interpretations of our measurements difficult. However, these previously reported levels are on the order of the observed enhancement factors in Fig. 5, and thus we must assume that ships have the potential to contribute significantly above the ambient background.

**Table 2:** Published polar and sub-polar INP concentrations. Continuous Flow Diffusion Chamber (CFDC) techniques are used to study ice nucleation *in situ*.

Location	Campaign	Average INP/L	T (C) & S*i* conditions	Technique/Nucleation Mode	Study
Arctic	M-PACE	≈0.7	−6° to −28°	CFDC	Prenni *et al*.^48^
Arctic	ISCAC	0.1–10	−10% ≤ SS_w_ ≤ 10%	CFDC	McFarquhar *et al*.^49^
Arctic	Aircraft	0–100 s mostly ≤1.0	−10° to −30°	CFDC	Rogers *et al*.^50^
Scandinavia	waveclouds	≤100	−12° to −40°	CFDC	Field *et al*.^51^
Arctic	ASCOS cruise	below resolution	−12° to −40°	CCN	Martin *et al*.^52^

Furthermore, as the climate changes, shipping in the Arctic is already increasing dramatically. In addition to the up to 50% fuel savings that could potentially be achieved on Europe to Asia trading routes, reductions in sea ice open the Arctic to more oil, mineral, and gas exploration. Predictions suggest that as much as 2% and 5% of global sea trade could be shifted to the Arctic in 2030 and 2050 respectively. This represents an increase in annual fuel consumption in the Arctic (an extended Arctic Monitoring and Assessment Programme region as defined by Peters *et al*.^27^) of 2.9 to 5.2 megatonnes per annum^27^. Using the calculated emission factors from this study (≈2 × 10^7^ INP(kg fuel)^−1^, Fig. 6), this suggests that over the next decades shipping may account for 10^16^ to 10^17^ new Arctic INP yr^−1^. Although, dispersed over an entire region the signal may remain small it is important to note that such emissions will be point (or line) contributions, with much greater average intensities over channels of shipping traffic. Others have already shown that aerosol emissions from ships can, for example, lead to lightning enhancement in narrow bands above busy traffic channels^42^. In particular mixed-phase Arctic stratiform clouds have significant radiative feedbacks and can be widespread and persistent^43^. Whether the delicate balance would be maintained with the boundary layer injection of nucleating particles is a complicated calculus related to surface coupling and cloud feedbacks (cf. Shupe *et al*.^44^).

Satellite observations of Arctic mixed-phase clouds show that ice water content and surface precipitation are influenced and potentially increased by shipping^21^. Mixed-phase cloud modeling also suggests that even at low concentrations (${\mathscr{O}}\mathrm{[1]}$ INP L^−1^) INP recycling that occurs when cloud particles evaporate and/or sublimate near the cloud base, and the nuclei are thus free to be reutilized, is sufficient to maintain ice production and therefore regulate liquid water content over multiple days^45^. The Possner *et al*.^32^ study has tried to directly assess some of the competing microphysical effects using idealized large eddy simulations, which resolve the large scale turbulent structures within clouds, to perform a case study based upon observations of single-layer mixed phase stratus with inserted ship emissions, including INP^32^. They find that microphysical feedbacks and cloud response are sensitive to the ice phase in non-obvious ways. The manifestation of cloud radiative and precipitation response to ship-perturbations is sensitive to background conditions and INP recycling. Furthermore, previously identified warm-phase stratus responses to emission’s perturbations may be compensated for, or diminished by, additional feedbacks involving the ice phase. For example, in warm-phase stratocumulus the cloud radiative effect of ship emissions has previously been determined to result from changes in cloud condensation nuclei increasing cloud optical thickness due to decreases in cloud droplet size and corresponding increases in liquid water path^46^. That effect can be somewhat mitigated when the ice phase helps to stabilize the cloud layer.

In summary, the FRIDGE electrostatic deposition and wafer analysis system for INPs has been used to collect and study the particles from ship emissions outside of the Port of Gothenburg. The ship emissions are observed to more than double the ice nucleating capability of the background aerosol. Given the boreal location of Gothenburg, Sweden and low ambient INP concentrations, the observations can be taken as an indication of potential impact for ships in the Arctic and/or other pristine aerosol regions. Although the Arctic remains isolated and remote it is currently in a period of unprecedented climate and ecosystem change, and much of the change is Arctic response to global forcing^47^. It is therefore important to consider what new effects will result due to anthropogenic encroachment, and as once distant influences become local drivers.
